# Exploring the potential of genetic analysis in historical blood spots for patients with iodine-deficient goiter and thyroid carcinomas in Switzerland and Germany (1929–1989)

**DOI:** 10.1186/s12920-024-01947-y

**Published:** 2024-06-28

**Authors:** Janine Schulte, Gerhard Hotz, Gabor Szinnai, Emanuel Christ, Gaspare Foderà, Karl Krüsi, Peter Nussberger, Sarah Kron, Iris Schulz

**Affiliations:** 1 Health Department Basel-Stadt, Institute of Forensic Medicine, University Basel, Pestalozzistrasse 22, Basel, 4056 Switzerland; 2 Natural History Museum Basel, Augustinergasse 2, Basel, 4001 Switzerland; 3 Pediatric Endocrinology/Diabetology, University Children’s Hospital Basel UKBB, University of Basel, Basel, 4056 Switzerland; 4 Division of Endocrinology, Diabetology, Metabolism and Center of Endocrine and Neuroendocrine Tumors, University Hospital Basel, Basel, 4056 Switzerland; 5 Documentation Center, Municipal Administration, Riehen, 4125 Switzerland; 6 Surgery Department, Adullam Hospital, Riehen, 4125 Switzerland

**Keywords:** Feasibility study, Goiter imprints, Historical dried blood spots, Genetic analysis, DNA quantity and quality

## Abstract

Iodine deficiency-induced goiter continues to be a global public health concern, with varying manifestations based on geography, patient’s age, and sex. To gain insights into clinical occurrences, a retrospective study analyzed medical records from patients with iodine deficiency-induced goiter or thyroid cancer who underwent surgery at the Community Hospital in Riehen, Switzerland, between 1929 and 1989. Despite today’s adequate iodine supplementation, a significant risk for iodine-independent goiter remains in Switzerland, suggesting that genetic factors, among others, might be involved. Thus, a pilot study exploring the feasibility of genetic analysis of blood spots from these medical records was conducted to investigate and enhance the understanding of goiter development, potentially identify genetic variations, and explore the influence of dietary habits and other environmental stimuli on the disease.

Blood prints from goiter patients’ enlarged organs were collected per decade from medical records. These prints had been made by pressing, drawing, or tracing (i.e., pressed and drawn) the removed organs onto paper sheets. DNA analysis revealed that its yields varied more between the prints than between years. A considerable proportion of the samples exhibited substantial DNA degradation unrelated to sample collection time and DNA mixtures of different contributors. Thus, each goiter imprint must be individually evaluated and cannot be used to predict the success rate of genetic analysis in general. Collecting a large sample or the entire blood ablation for genetic analysis is recommended to mitigate potential insufficient DNA quantities. Researchers should also consider degradation and external biological compounds’ impact on the genetic analysis of interest, with the dominant contributor anticipated to originate from the patient’s blood.

## Highlights

DNA can be extracted from paper-derived historical blood spots using a commercial kit.

Strong fluctuation in DNA quantity and quality over and within decades due to storage and samples’ age.

DNA mixtures are to be expected due to the handling of patient files by multiple individuals.

Analysis of blood ablations should be considered individually and holistically.

## Introduction

Iodine is an essential trace element for synthesizing thyroid hormones and properly functioning the normal thyroid gland [1–3]. Thus, inadequate nutrient supply can cause goiter development, characterized by an abnormal enlargement of the thyroid glands [1, 4]. Additionally, insufficient thyroid hormone levels during fetal development and childhood can impair growth, mental disabilities, hearing loss, and other neurological deficits [3, 5].

Iodine is distributed throughout the planet’s ecosystem, but its occurrence and distribution can vary across different regions, while the majority is found in the oceans, atmosphere, and soil. However, some areas may experience iodine deficiency in soils and groundwater, which, in turn, can decrease dietary intake [2, 4, 6, 7]. While the recommended daily consumption of iodine for healthy adults is between 150 and 250 µg/day [3, 6–8], the primary approach to managing iodine deficiency disorders (IDD) is through salt iodization endorsed by the World Health Assembly in 1993. Salt was chosen as a vehicle as it is widely consumed, and iodization is relatively low-cost. In the early 1960s, only a few nations implemented IDD control initiatives, primarily in the United States and Europe. Over the past thirty years, significant progress has been made in increasing access to iodized salt and reducing iodine insufficiency in most global regions. Despite these efforts, iodine insufficiency remains a significant concern for public health, particularly in Europe [9].

Historically, the Swiss population faced moderate to severe iodine deficiency, resulting in a higher incidence of goiter and cretinism [8, 10]. The latter describes a more severe condition characterized by profound and irreversible physical and mental impairments (i.e., stunted growth, intellectual disability, delayed development, and physical deformities) caused by extensive and prolonged iodine deficiency during early development [1, 11]. In 1923, a hospitalization rate of 0.1% was observed among the population of Riehen due to cretinism-induced inability to self-care. A survey conducted in 1975 revealed that individuals aged 60–79 had a goiter prevalence three times higher (60%) compared to individuals aged 20–39 [8]. As Switzerland was an early adopter of iodized salt in 1922 to address and prevent iodine deficiency, iodized salt was widely accessible throughout the country by 1952 [2, 3, 10]. However, in response to variations in salt consumption, the concentration of iodine in salt, in the form of potassium iodide, was gradually increased several times, from 3.75 mg/kg in 1952 to 7.5 mg/kg in 1962, 15 mg/kg in 1980, 20 mg/kg in 1998, and 25 mg/kg in 2014. While doing so, the prevalence of goiter has steadily declined, indicating that the treatment was successful and suggesting an iodization shortage, notably during the earlier phases [2, 3, 10, 12].

Although dietary iodine deficiency is the most common cause of goiter, the multifactorial nature of the disease and the postulated complex interaction between environmental and endogenous factors, such as genetic, metabolic, hormonal, and immune system factors, as well as, inflammation, neoplastic processes, and age, is nowadays undisputed [1, 13–24]. While endemic goiter occurs commonly in iodine deficiency areas, the environment alone cannot solely account for the goiter etiology. In contrast, the development of the less prevalent sporadic or iodine-non-related goiter is more influenced by individual factors, whereby an interplay with environmental exposures (e.g., lifestyle choices) cannot be ruled out. This means that not every individual develops a goiter, and despite iodine supplementation efforts, not all persons are spared from goiter development [13–15, 25]. Thus, genetic mutations (i.e., gene variations) [13–16, 26–28] or different gene expression patterns by small non-coding RNA (e.g., microRNA) expression profiles or epigenetically changes (i.e., DNA methylation, histone modification) [16, 29, 30] may predispose individuals to thyroid cancer and goiter, also supported by twin and family studies [13, 14, 25]. With research aiming to further identify and understand the role of (epi-)genetic, predictive markers in the disease’s development, progression, and therapeutic intervention, a polygenic character is assumed [15].

The Documentation Center in Riehen houses a substantial and well-maintained archive of medical records related to iodine-deficient goiter and thyroid carcinomas. These records span from 1929 to 1989, with approximately 10% containing blood ablations derived from surgically removed goiters. The center served as a catchment area that includes regions of Switzerland and southern Germany [31]. The iodine fortification programs in both countries were implemented at different times, providing an opportunity to gain insights and compare information on goiter incidences and the effectiveness of iodine fortification strategies based on recorded anamnestic, clinical, intraoperative, postoperative, histological and demographic parameters in two geographically close but administratively, socioeconomically, culturally, and dietary distinct areas [2, 32, 33]. For this, consistent documentation and standardized transcription of medical records, surgical interventions, and histological findings of nearly all patients from 1929 to 1989 are available, allowing for longitudinal, retrospective epidemiological, and potentially deeper genetic inquiries from bloodstains, providing sufficient DNA quantity and quality, which will be explored in this feasibility study.

Hence, a pilot study was designed, conducting a preliminary assessment of the overall feasibility of potentially upcoming genetic analysis using historical blood spots from medical records of individuals diagnosed with thyroid cancer and iodine-deficient goiters, providing a solid basis for addressing possible genetically amenable inquiries. In addition, this initial investigation allows the detection and identification of anticipated issues, such as genetic degradation or the presence of DNA mixtures, while also potentially uncovering novel difficulties. By revealing these challenges at an early stage, resources and outcome-oriented decisions can be made for subsequent studies, while appropriate modifications can be implemented to refine the approach for subsequent analysis adequately.

## Materials and methods

### Feasibility study

For the feasibility study, 1’000 medical records of patients who underwent goiter surgery between 1929 and 1989 at the Riehen Hospital were available. These records contained outlines of the goiter impressions used for the investigation. Goiter blood prints were made by pressing, drawing, or tracing the removed organs onto paper sheets. Ten samples with assumed blood marks were analyzed from each decade (i.e., 1929 to 1979) following the criteria outlined in the “Sample Collection” section and using a random selection process from patient files. The study was performed with an approved ethics application by the Ethikkommission Nordwest- und Zentralschweiz (EKNZ).

### Sample collection

**Fig. 1:**
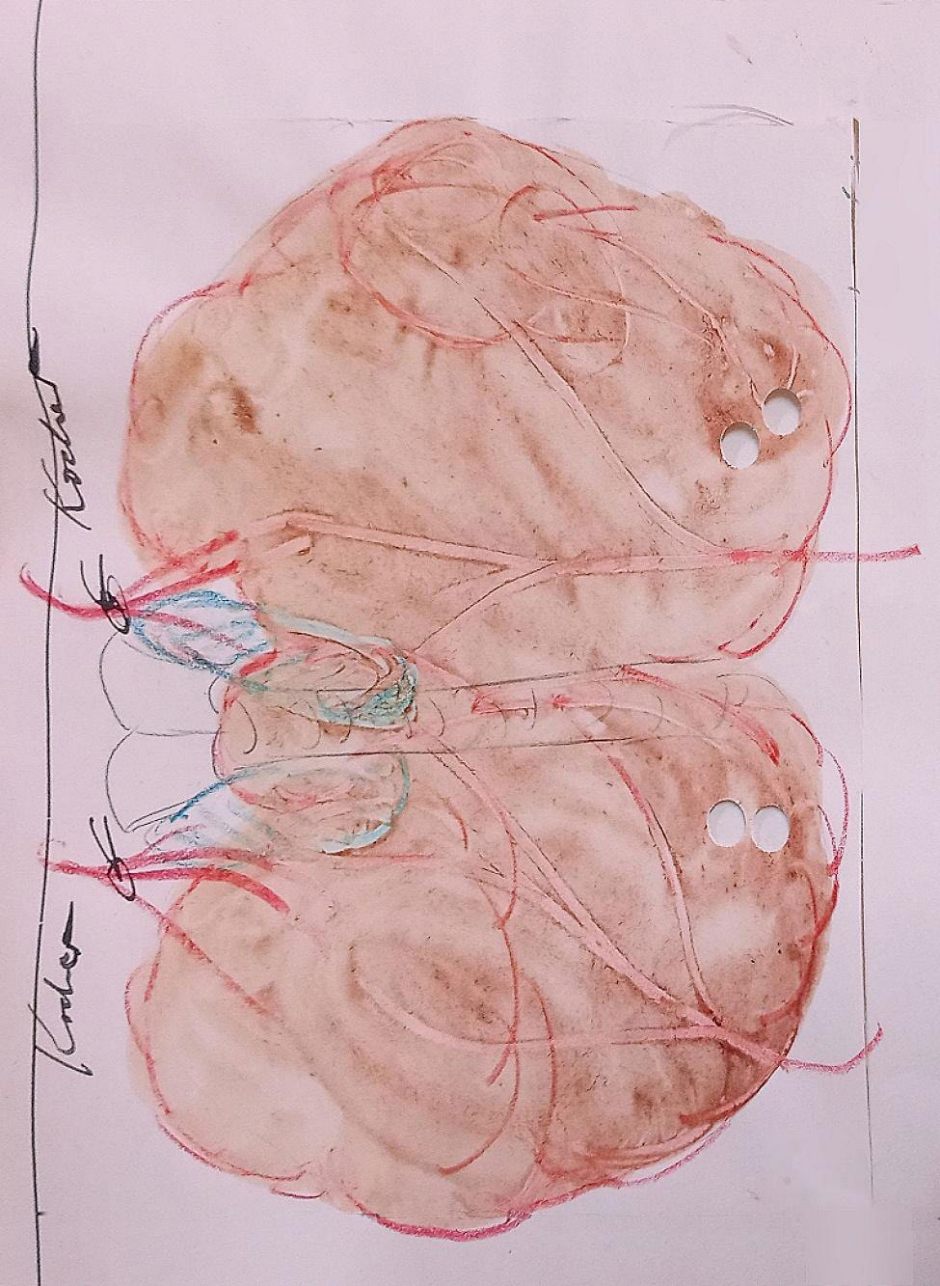
Goiter imprint. Exemplary goiter impression from a patient file. The tissue removed by surgery was imprinted and traced, while the corresponding spots were punched out using a hole puncher

For genetic analysis, patient files were pre-screened according to the following criteria: (1) sufficiently large imprint (approximately half a page) with assumed blood stains, (2) no handwriting or later typewriter print on the backside of the imprint, (3) presence of multiple blood deposits (as dark as possible), (4) minimal or no drawings within the impression. Figure 1 depicts an example of the imprints being investigated.

**Fig. 2:**
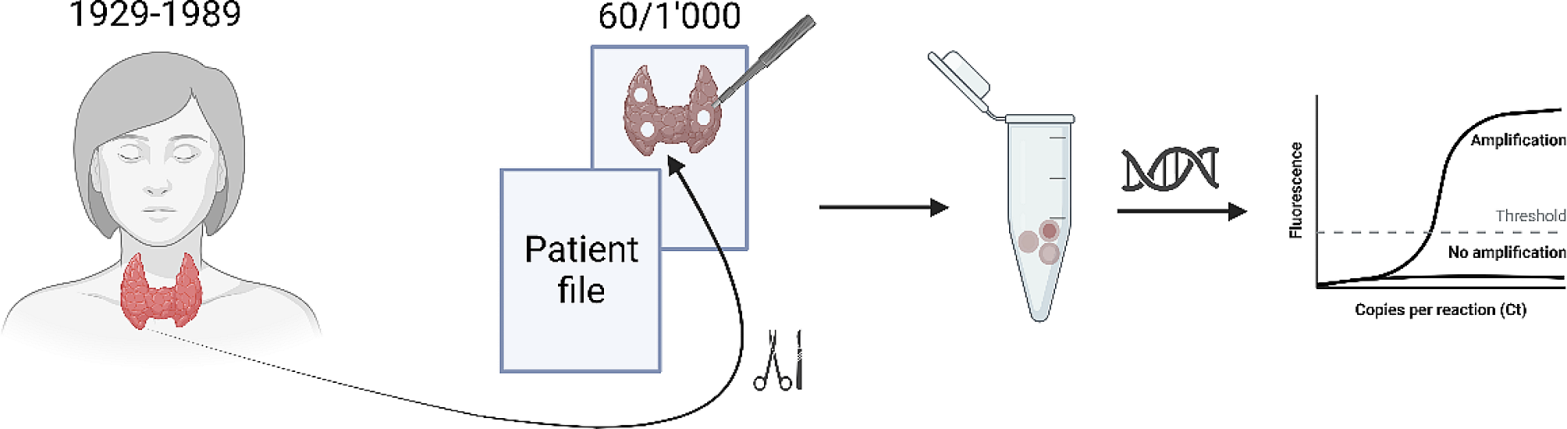
Sample Collection. Randomly selected blood imprints meeting the outlined criteria were extracted and quantified for each of the decades 1929, 1939, 1949, 1959, 1969, and 1979 (*n* = 10 per decade); created by Biorender.com [34]

Ten specimens were randomly collected from the initial two years of each decade. Three replicate samples of equal size were obtained using a single-hole puncher and subsequently stored in 1.5 mL tubes under light-protected conditions at ambient temperature until further analysis (Fig. 2).

### Genetic analysis

The DNA obtained from dried blood spots of the medical records was isolated using the QIAamp^®^ DNA Investigator Kit (QIAGEN N.V., Venlo, The Netherlands) and eluted in 50 µL elution buffer. The kit facilitates the purification of genomic DNA from various forensically relevant biological materials (e.g., blood, saliva, sperm) found on diverse substrates (e.g., paper, textiles, stones) and collected by swabbing, taping, or cutting. The kit uses QIAamp MinElute spin columns to purify high-quality DNA, allowing optimal performance in subsequent quantitative polymerase chain reaction (qPCR) and other downstream analyses [35]. The quantification of DNA was carried out in triplicates using the Powerquant^®^ kit (Promega Corporation, Madison, WI, USA) on an Applied Biosystems™ 7500 Real Time PCR System (Thermo Fisher Scientific, Waltham, MA, USA) following the manufacturers’ protocols [36, 37], except for the internally validated reaction in half volume. The kit integrates multicopy quantification and an internal PCR control (IPC) into a single 5-dye qPCR assay that measures the total amount of human and human male DNA while identifying the presence of PCR inhibitors and DNA degradation ( [38], Table 1). Further, the Powerquant^®^ kit was selected from other established real-time quantification kits due to its reliable performance on challenging forensic samples with minute DNA amounts, often with low quality [39].

**Table 1:** Overview of the PowerQuant primers and probes for DNA quantification using the PowerQuant^®^ System. Data from the four dye channels are normalized to the passive reference dye, CXR dye (adopted from [37])

Target	Amplicon (bp)	Labeled probe	Features	Purpose
Autosomal	84	FAM	High sensitivity, robust to inhibitors, and less affected by degradation	Detects total human DNA
Y-Chromosomal	81 and 136	CAL Fluor^®^ Gold 540	Increased sensitivity for male DNA, reduces copy number variation effects	Detects human male DNA
Autosomal	294	Quasar 670	Same proprietary locus as the autosomal target but a larger amplicon	Detects degradation
Artificial	435	TMR	Amplifies an internal PCR control (IPC) as an introduced novel template, the longest amplicon	Detects inhibitors

The ratio of DNA concentrations determined with the autosomal and degradation targets ([Auto]/[Deg] ratio) can be used to evaluate the degree of degradation, while the IPC amplification performance is used to identify inhibitors in the sample. The software calculates the difference in the quantification cycle (Cq) values for the IPC in (1) an unknown sample and the IPC in (2) the closest DNA standard of the standard curve (i.e., IPC shift). This data can be used to make informed choices regarding the purification of samples, the dilution of DNA samples, and the ideal template volume to include in amplifying regions of interest in the human genome [37, 38].

### Data analysis

With the PowerQuant^®^ System, the quantity of autosomal and Y-chromosomal DNA was measured in ng/µL, while the quality was assessed by the degradation index (DI) and IPC shift. Table 2 gives the interpretation guidelines for the occurrence of degradation and/or inhibitors in the unknown sample.

**Table 2:** Interpretation guidance for degradation or inhibition, potentially occurring in quantified samples using the PowerQuant^®^ System. The thresholds for degradation or inhibition were adapted according to internal validation studies with a threshold value of 10 for [Auto]/[Deg] and 5 for the IPC shift. “No Deg Cq” = no degradation quantification cycle [37]

[Auto]/[Deg] Ratio	IPC Shift	Interpretation
< Threshold	irrelevant	Unlikely to be degraded
> Threshold or No deg Cq	< Threshold	Likely to be degraded, without PCR inhibitors
> Threshold or No deg Cq	> Threshold	Likely to contain inhibitors, may have degradation

The R statistical software [40] was used for data visualization and descriptive statistics. The statistical analysis employed the Kruskal-Wallis-test with Dunn’s post hoc test for multiple, pairwise comparisons to identify the different groups. The tests were performed with a significance level of 5%.

## Results

### DNA quantity

**Fig. 3:**
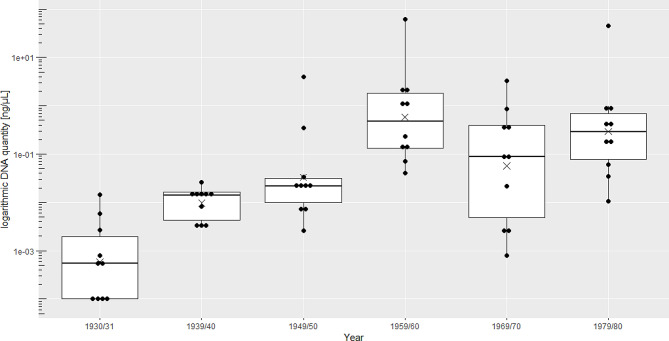
Logarithmic DNA quantities (ng/µL) of the 10 respective goiter impressions per decade. Statistical significance was found (Kruskal-Wallis-Test, *p* < 0.05) between the first decade compared to 1959/60, 1969/70, 1979/80, and between 1939/40 and 1959/40, but not between the last four groups. Each point represents the mean of three independently performed quantitative PCRs; “X” marks mean DNA values

The samples collected during the initial two decades exhibited only minute amounts of extractable DNA, with concentrations ranging from a more theoretical DNA amount of 0.0001 ng/µL to 0.0259 ng/µL, roughly equivalent to 4 cell nuclei (mean = 0.007 ng/µL, median = 0.003 ng/µL, Fig. 3). On the other hand, samples from the last four decades (Fig. 3) exhibited a notable amount of DNA, with a wide distribution range from 0.0008 ng/µL to 61.21 ng/µL (mean = 3.16 ng/µL, median = 0.14 ng/µL). Since 1982, there has been no documentation of goiter imprints in the patient records of the community hospital; thus, there were no representative samples for the last decade available.

Determining the combined quantity of amplifiable autosomal and Y-chromosomal DNA provides insights into the presence of male, female, or mixed DNA components in the sample, with the latter only detectable for different genders. Over 50% of the specimens exclusively exhibit DNA from females, with the possibility of same-gender mixtures. A male-female DNA mixture was observed in 25% of the samples. Male DNA, or male-male mixtures, were detected in around 7% of the samples, while for the remaining samples, the triplicate quantification was inconclusive due to unclear or unreliable results.

### DNA quality

Table 3 displays the degradation indices obtained from the samples, which varied from 18 (moderate degradation) to ≥ 3’000 (severe degradation); however, [Auto]/[Deg] ratios may not be reliable in samples with low DNA concentrations (i.e., less than 1pg/µl) due to stochastic effects [37]. Samples with no detected value for the degradation target (i.e., undetermined = No Deg Cq) indicate that the sample is either present in too low amounts for accurate measurement or severely damaged. In total, 4 samples showed inconclusive and thus not evaluable results within triplicate analysis (= NA). IPC shifts of each sample were below the established threshold value of 5. In combination with the [Auto]/[Deg] ratios for the last four decades, it can be estimated that the samples depicting “No Deg Cq” are severely degraded but do not contain PCR inhibitors.

**Table 3:** Mean degradation indices (DI) per sample over the decades spanning from 1930 to 1980

Sample	Year					
	1930/1931	1939/1940	1949/1950	1959/1960	1969/1970	1979/1980
1	NA*	No Deg Cq	21	325	25	278
2	No Deg Cq	No Deg Cq	No Deg Cq	23	No Deg Cq	NA**
3	No Deg Cq	No Deg Cq	213	989	767	18
4	NA*	No Deg Cq	132	387	85	586
5	No Deg Cq	No Deg Cq	No Deg Cq	668	287	934
6	No Deg Cq	No Deg Cq	No Deg Cq	31	1091	321
7	No Deg Cq	No Deg Cq	NA**	45	3130	312
8	No Deg Cq	No Deg Cq	No Deg Cq	136	483	No Deg Cq
9	19	No Deg Cq	NA**	447	No Deg Cq	24
10	No Deg Cq	No Deg Cq	No Deg Cq	208	No Deg Cq	675

*Triplicate analysis showing inconclusive results (not applicable = NA); **Two values show “No Deq Cq” and one value shows strong degradation (231, 297 and 362)

## Discussion

### DNA quantity

The low DNA levels, despite the presence of blood traces with expected high DNA amounts, can be attributed to influencing factors such as the samples’ age along with unfavorable, uncontrolled storage conditions. Finding DNA below detectable quantification thresholds is not surprising and is in line with various studies working on stored and degraded dried blood stains [41–47] or exploring the limits of detection [48, 49]. With respect to subsequent downstream analysis, it is essential to note that a low quantification result does not necessarily result in a profiling failure using standard forensic amplification kits, which, however, remains to be investigated for further in-depth downstream genetic analyses. For example, research demonstrated that DNA profiles could be generated from samples with literally no DNA quantities [39, 50]. Moreover, the alleged imprints of the medical records before 1940 posed a challenge in differentiation from the paper discoloration that occurs with the age of the patient’s files and/or medical illustrations that inherently lack DNA content.

Over 50% of the specimens exclusively exhibit DNA from females, which is not unexpected considering the higher prevalence of goiter in females than in males [51–54]. A meta-analysis by Malboosbaf et al. [51] suggested that the release of reproductive hormones during puberty might contribute to this gender difference, as testosterone, unlike estrogen, might inhibit thyroid enlargement. In these mixtures, the presence of both genders might be due to various factors, including the patient’s biological material, potential contamination from the surgeon imprinting the organ, and handling of the patient’s documents by others, leading to the transfer of their DNA [55, 56]. It is important to note that the files were not handled sterile or DNA-protective, which will likely have contributed to the presence of mixed DNA traces from unknown sources, numbers of contributors, and different genetic contributions. However, given that the study was intended as a feasibility assessment, it was not sensible (and not permitted) to access personal data for comparison of the DNA information with the actual gender and number of subjects.

### DNA quality

The PowerQuant^®^ System enables the quantification of autosomal DNA and provides insights into the extent of DNA degradation and inhibition in the analyzed samples. Especially for the first decades, no DI value could be determined as no or only small amounts of DNA were quantified. Concerning the latter, the deterioration level of individual samples can be influenced by various factors such as sample age or storage time and conditions [57, 58], substrate (e.g., paper type, composition, and deterioration [59–61]), and environmental factors (e.g., humidity, temperature, and light exposure [57, 58]). The lack of inhibition is remarkable, as the imprints were manipulated with pen ablations and possibly other unknown interventions. However, care was taken to ensure that the impressions did not have many inscriptions to preserve the documentation of these historical patient files. Therefore, no whole impressions were used for the pilot study. The shown variations in both the amount and quality of DNA obtained from tissue ablations may be attributed to distinct printing methodologies, including the magnitude and duration of pressure applied, the length of time for drying, hindering factors such as pen residues, and undoubtedly the subsequent manipulation of the files by external entities (and their DNA requisitions).

In terms of sample age and storage condition, it has already been reported that DNA in blood stains can generally remain stable for several weeks and months at ambient temperature (i.e., room temperature) or even years using stabilizing agents [49, 62–65]. As deposited biological material is subject to endogenic enzymes, microbial processes, and chemical reactions causing damage to cellular material and thus the degradation of nucleic acids, appropriate storage (e.g., temperature consistency and frigidity) remains crucial [66–68]. In contrast, air humidity was predicted to affect cell condition mainly but not directly DNA stability [69]. Regrettably, prior to 2012, the patient records were stored without proper protection in the attic of the municipal administration, where natural temperature fluctuations likely affected the integrity of the biological specimens. Moreover, the latest sample analyzed was still over 40 years old.

From a forensic standpoint, it remains controversial whether the majority of the detected DNA quantities are appropriate for additional analyses, including classical short tandem repeat (STR) analysis utilizing multiplex polymerase chain reaction (PCR) or using single nucleotide polymorphisms (SNPs) and next-generation sequencing (NGS) [70–72]. Recent advances in the sensitivity of techniques and reagents have enabled DNA profiling from quantities as low as contact traces [50, 73] or even individual cells [74]. Further, NGS has successfully analyzed degraded samples from human remains and artificially degraded DNA through UV treatment [75]. However, as indicated through quantification, mixtures of biological material are likely to occur, as it reflects various individuals’ handling of patient records over time [55, 56, 76]. Additionally, the partially poor DNA quality in the blood spots, characterized by degradation, may pose extra challenges for further intended genetic inquiries.

Although evaluating the real-time PCR quantification results still requires great care, it has already been shown that the quantification assay enables a correlation between the real-time quantification values and the STR genotyping results [39]. Through internal validation, a DI of 10 is classified as severely impaired. Insufficient DNA quantity and quality can affect the genotyping outcome and cause, for example, genetic artifacts, dropouts, and imbalanced peaks in the STR profile [44, 58, 77–79]. These challenges can complicate the accurate interpretation of DNA profiles. Conversely, for the samples with sufficient amounts of DNA, potential mixtures are expected to consist primarily of patient DNA, which comes from blood material with a comparatively higher concentration than the smaller amounts from contact traces, e.g., epithelial cells, which are left behind when touching the imprints. In addition, touch DNA is likely more susceptible to degradation and declines more rapidly over time compared to the patient’s blood samples [79–81]. As STR profiling would have required enhanced ethics approval, given the inclusion of personalized patient data, an initial assessment of the usability of historical bloodstains was performed by DNA quantification.

## Conclusion

The feasibility study showed that DNA extraction and quantification could generally be performed on historical blood imprints from medical reports of patients with iodine-deficient goiter and thyroid carcinomas. However, a considerable portion of the unprotected stored samples exhibited significant challenges, such as DNA quantity variations, substantial DNA degradation, and DNA mixtures. The latter is likely to contain a major contribution from the patient’s DNA (blood) and minor, unidentifiable proportions of the contact DNA left behind when handling the imprints or documents. Consequently, a general success rate cannot be reliably predicted. Instead, each goiter imprint must be evaluated case-by-case for further genetic analyses considering its individual condition but also available resources and desired outcomes. To counteract the effects of low DNA quantities, it would be sensible to use the more recent documents, larger sample material, or the entire imprint whenever possible, while acknowledging potentially increased inhibition.

## Data Availability

The datasets used and/or analysed during the current study available from the corresponding author on reasonable request.
